# Brain Response to a Knee Proprioception Task Among Persons With Anterior Cruciate Ligament Reconstruction and Controls

**DOI:** 10.3389/fnhum.2022.841874

**Published:** 2022-03-22

**Authors:** Andrew Strong, Helena Grip, Carl-Johan Boraxbekk, Jonas Selling, Charlotte K. Häger

**Affiliations:** ^1^Department of Community Medicine and Rehabilitation, Physiotherapy, Umeå University, Umeå, Sweden; ^2^Department of Radiation Sciences, Umeå University, Umeå, Sweden; ^3^Danish Research Centre for Magnetic Resonance (DRCMR), Centre for Functional and Diagnostic Imaging and Research, Copenhagen University Hospital Hvidovre, Copenhagen, Denmark; ^4^Umeå Center for Functional Brain Imaging (UFBI), Umeå University, Umeå, Sweden; ^5^Institute of Sports Medicine Copenhagen (ISMC), Copenhagen University Hospital Bispebjerg, Copenhagen, Denmark

**Keywords:** anterior cruciate ligament, anterior cruciate ligament reconstruction, knee, rehabilitation, position sense, magnetic resonance imaging, neuronal plasticity

## Abstract

Knee proprioception deficits and neuroplasticity have been indicated following injury to the anterior cruciate ligament (ACL). Evidence is, however, scarce regarding brain response to knee proprioception tasks and the impact of ACL injury. This study aimed to identify brain regions associated with the proprioceptive sense of joint position at the knee and whether the related brain response of individuals with ACL reconstruction differed from that of asymptomatic controls. Twenty-one persons with unilateral ACL reconstruction (mean 23 months post-surgery) of either the right (*n* = 10) or left (*n* = 11) knee, as well as 19 controls (CTRL) matched for sex, age, height, weight and current activity level, performed a knee joint position sense (JPS) test during simultaneous functional magnetic resonance imaging (fMRI). Integrated motion capture provided real-time knee kinematics to activate test instructions, as well as accurate knee angles for JPS outcomes. Recruited brain regions during knee angle reproduction included somatosensory cortices, prefrontal cortex and insula. Neither brain response nor JPS errors differed between groups, but across groups significant correlations revealed that greater errors were associated with greater ipsilateral response in the anterior cingulate (*r* = 0.476, *P* = 0.009), supramarginal gyrus (*r* = 0.395, *P* = 0.034) and insula (*r* = 0.474, *P* = 0.008). This is the first study to capture brain response using fMRI in relation to quantifiable knee JPS. Activated brain regions have previously been associated with sensorimotor processes, body schema and interoception. Our innovative paradigm can help to guide future research investigating brain response to lower limb proprioception.

## Introduction

Rupture of the anterior cruciate ligament (ACL) is a common knee injury among athletic populations (Majewski et al., 2006), with a reported 30% rate of secondary ACL injury up to 15 years post-reconstruction (Leys et al., 2012) and a four times higher risk for knee osteoarthritis (Poulsen et al., 2019). Evidence further indicates that individuals with ACL reconstruction (ACLR) have lesser bilateral corticospinal excitability than those without injury, which may have a detrimental effect on muscle recovery (Rush et al., 2021). The initial trauma and potential surgical reconstruction causes loss of neural elements such as Golgi tendon organ-like receptors (Cabuk et al., 2022). These receptors contribute with afferent information to the central nervous system (CNS) regarding proprioceptive sensations such as movement and position (Dhillon et al., 2012). Of the proprioceptive senses, joint position sense (JPS) is one of the most commonly tested, typically involving the passive or active reproduction of joint angles with the confounding sense of vision occluded (Hillier et al., 2015). Outcomes are based on the difference in degrees between the target and reproduced angles, thus reflecting the kinematic errors. Identifying deficient knee JPS is believed important as it may contribute to errors in coordination which can subsequently expose the joint to positions deemed to increase the risk for injury (Needle et al., 2017). Meta-analyses have found significantly greater knee JPS errors for ACL-injured knees compared to both the contralateral non-injured knees of the same individuals (Relph et al., 2014; Kim et al., 2017; Strong et al., 2021a) and to those of asymptomatic persons (Relph et al., 2014; Strong et al., 2021a). The clinical significance of these findings is, however, unclear given the small absolute differences of < 1° knee flexion angle. Existing JPS tests have also been criticized for lacking reliability and validity (Hillier et al., 2015). Therefore, despite the belief that neurosensory information and knee proprioception may be impaired following ACL injury (Nyland et al., 2017), simply comparing knee JPS errors may be insufficient in detecting intricate alterations to the CNS (Baumeister et al., 2008). Thus, although knee JPS tests appear not to reveal clinically significant levels of deficient proprioception following ACL injury, reorganization of the CNS has nonetheless been hypothesized (Kapreli and Athanasopoulos, 2006). It is specifically believed that effects on the somatosensory and motor cortices may lead to proprioception-related deficits in preparatory and reactive motor abilities, consequently contributing to the increased risk for secondary injury (Needle et al., 2017).

Brain response associated with lower limb proprioceptive acuity is unclear. Callaghan et al. (2012) implemented a single-joint knee JPS task during functional magnetic resonance imaging (fMRI) among asymptomatic controls and found greater blood-oxygen-level-dependent (BOLD) response in the supplementary motor area, ventral tegmental area, primary sensory cortex, cerebellum and precentral gyrus compared to a similar movement without angle reproduction. However, no outcomes of the JPS test were recorded, the sample size was small (*n* = 8) and the authors recommended a multi-joint movement to better represent normal functional tasks. In the same study, patellar taping, hypothesized to increase proprioceptive input, decreased response in the anterior cingulate and cerebellum. Based on a similar hypothesis, Thijs et al. (2010) investigated the influence of a knee brace and knee sleeve on brain response during lower limb multi-joint movements among asymptomatic individuals. Compared with no garment, greater response was found in the frontal lobe and paracentral lobule for the knee brace, as well as for the parietal lobe and superior parietal lobule for the knee sleeve. The combined findings of these studies indicate that changes to afferent information at the knee alters brain response during lower limb movements.

Injury to the ACL is believed to cause adaptations to the CNS (Needle et al., 2017). A recent scoping review of the topic by Neto et al. (2019) indeed found evidence for greater brain response compared to controls in mainly cortical areas associated with sensory and motor processes. One electroencephalography (EEG) study by Baumeister et al. (2008) incorporated a test of knee JPS and found greater frontal Theta-power for individuals with ACLR compared to controls. This response was believed to generate from the anterior cingulate cortex due to attentional demands and task complexity. Correlations for both groups additionally showed a reduction of JPS errors over time together with decreased processing in the parietal somatosensory cortex. However, EEG is limited in providing exact locations of the electrical sources from scalp recordings, while fMRI provides a higher spatial resolution (Ritter and Villringer, 2006). The only studies which used fMRI were those by Kapreli et al. (2009) and Grooms et al. (2017) who used similar task designs of single-joint knee flexion and extension. Both studies found less cerebellum activation and greater activation of secondary somatosensory areas for their ACL groups compared to their respective control groups, but also some inconsistent results for other regions. Differences between the ACL populations, such as treatment strategy, i.e., with or without reconstruction, and contrasting activity levels may have contributed to the divergent results. Grooms et al. (2017) suggested that multi-joint movements would improve the clinical applicability of future investigations. A more recent study thus included repetitive multi-joint heel slide movements and found that individuals with ACLR had greater response in areas associated with visual-spatial cognition and orientation compared with asymptomatic matched controls (Criss et al., 2020). It has, however, been recommended to improve the clinical applicability of such findings by increasing the motor control demands of such tasks by including, e.g., a proprioceptive goal-oriented element such as position matching (Grooms et al., 2017; Criss et al., 2020).

To summarize, brain response to proprioceptive tasks of the lower extremities remains unclear. Injury to the ACL may cause deficits to knee proprioception and related adaptations of the CNS. We have previously developed a supine knee JPS test which can be adapted for use in an fMRI setting (Strong et al., 2021b). Importantly, our standardized knee JPS test is unique for an fMRI setting in retaining otherwise common methods, whereby repetitions of knee angle memorization and subsequent reproduction are performed. Additionally, an integrated motion capture system not only provides test instructions to the participant based on their real-time kinematics, but is also used to identify knee movement phases for extraction of fMRI data during only relevant time periods. The kinematics further make possible the accurate reporting of angular errors to assess proprioceptive acuity and its potential association with response in specific brain regions. We therefore aimed to investigate the possibility of characterizing brain response using fMRI during simultaneous performance of a novel quantifiable knee JPS test among asymptomatic controls and individuals with unilateral ACLR. The specific research questions were: (1) Does our knee JPS test evoke a different brain response compared to a similar knee flexion movement without an angle reproduction task? (2) Is brain response different in persons with ACLR compared to asymptomatic persons during our knee JPS test? (3) Does brain response correlate to knee JPS test errors captured by kinematics? We hypothesized that our knee JPS test would recruit somatosensory and motor cortices more than during simple knee flexion and that individuals with ACLR would show greater response in such regions compared to asymptomatic persons. We further hypothesized that knee JPS errors would correlate with BOLD response in associated brain regions.

## Materials and Methods

### Participant Selection

For this cross-sectional study, participants were recruited from April 2017 to May 2019 using convenience sampling *via* orthopedic clinics, sports clubs, advertisements at the local University, social media and *via* word of mouth. Screening ensured the following eligibility criteria were met: aged 17–35 years, magnetic resonance imaging compliance, current Tegner activity score (Tegner and Lysholm, 1985) of at least 4, ability to understand either Swedish or English language, no known previous or ongoing injuries or diseases (other than ACLR and possible concomitant meniscus injury in the previous 5 years) that could affect the CNS or leg movements. Specific criteria for participants of the ACLR group required unilateral hamstring autograft reconstructive surgery performed 6 months to 5 years prior to testing, limited to only one ACL injury and subsequent surgery. All ACLR participants had to be cleared for full return to activity by their physical therapist. Asymptomatic controls were to be right-side dominant (leg preferred to kick a ball) and matched to ACLR participants with regard to sex, age, height, mass and current Tegner activity score. Our study is the first to incorporate this JPS paradigm during fMRI and thus data was not available to perform power calculations to estimate the required sample sizes. Considering previous similar research, one fMRI study comparing a different knee JPS task to a similar movement without a JPS task included eight healthy males (Callaghan et al., 2012). Previous fMRI studies comparing individuals with ACL injury to controls have included groups of either 15 (Grooms et al., 2017; Criss et al., 2020) or 17/18 (Kapreli et al., 2009), whereas a previous EEG study incorporating a knee JPS test included groups of 10/12 (Baumeister et al., 2008), respectively. Considering that our task design was calculated to result in fewer brain volumes than the aforementioned studies, we estimated that a pooled group of 40 participants would be required to investigate the brain regions recruited by our JPS test and 20 per group to examine potential differences in brain response between the ACLR and asymptomatic groups. The project was approved by the Regional Ethical Review Board in Umeå, Sweden (Dnr. 2015/67-31) and was performed in accordance with the relevant guidelines and regulations stated in the Declaration of Helsinki. All participants provided their written informed consent prior to participation.

### Procedures

Data collection occurred between June 2017 and May 2019. All participants completed the Marx Activity Scale (Marx et al., 2001) and the Tegner Activity Scale (Tegner and Lysholm, 1985). The following questionnaires were also completed by the ACLR participants: 2000 International Knee Documentation Committee Subjective Knee Form (IKDC; Irrgang et al., 2001); Lysholm Scale (Lysholm and Gillquist, 1982); and the Swedish version of the Tampa Scale of Kinesiophobia (TSK; Lundberg et al., 2004). Participants then performed a supine knee JPS test in the U-motion laboratory at Umeå University, Sweden to familiarize themselves with the task. Approximately 1 h later they performed the knee JPS test in an MRI scanner at the Umeå center for Functional Brain Imaging, University Hospital of Umeå, Sweden.

### Knee Joint Position Sense Test Protocol

**FIGURE 1:**
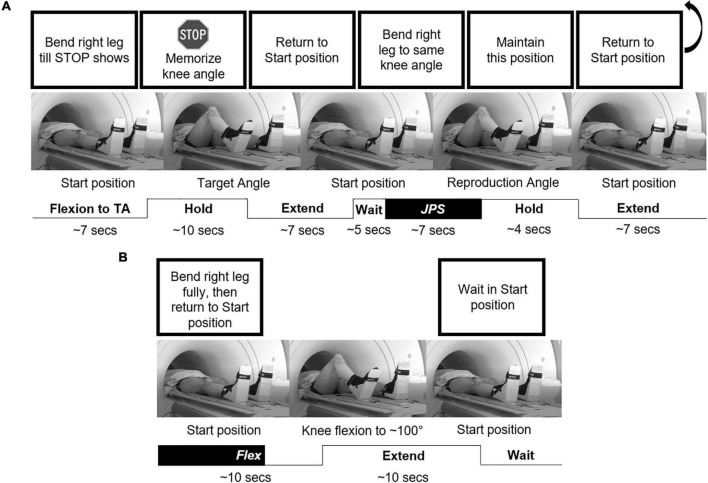
An illustration of the experimental setup. The individual is performing with their right leg **(A)** one repetition of the JPS test with a target angle of 65°, and **(B)** one repetition of the *Flex* condition. Example instructions shown at the top were displayed on a screen at the rear of the scanner visible to the participant *via* a tilted mirror attached to the head coil. BOLD response was measured during the highlighted “*JPS*” (from Start position to reproduction angle) and “*Flex*” (from Start position to 65° knee flexion) blocks, respectively, as well as for a “*Rest*” block (Start position for 15 s). TA, target angle.

A knee JPS test was specifically designed for fMRI compatibility by using a supine position, slow active movements and additional rest blocks. It was considered important that the test reflect those typically applied in the literature whereby target angle memorization is performed immediately prior to each attempt to reproduce the angle (Han et al., 2016). It was further important to assess brain response only during blocks for which proprioceptive signal processing was considered to be most pertinent for perception and movement control. This is in contrast to a previous study in which brain imaging analyses were performed without memorization prior to each reproduction attempt and during movement to a start position for which proprioception was less relevant and represented half of the brain images assessed (Callaghan et al., 2012). Standardized written test instructions were provided to each participant and any questions were answered. A three-camera motion capture system (Oqus MRI Qualisys AB, Gothenburg, Sweden, 120 Hz) provided real-time kinematic data for the lower limbs. Passive retro-reflective spherical markers were affixed with participants in standing on the skin overlying the greater trochanter and lateral epicondyle of each femur using skin-friendly double-sided adhesive tape. The greater trochanter markers were placed on sticks 56 mm in length (wand markers) to improve their visibility. Participants then lay supine with their feet in foot holders of a custom-made low-friction knee flexion/extension board (see Figure 1). Elastic bands with hook-and-loop fasteners secured each foot and lower shank to the foot holders to ensure a 90° ankle joint angle. A strap over the torso and cushions in the head coil limited head movement. Wand markers with 46 mm sticks were affixed on the lateral part of each foot holder in line with the lateral malleolus in the sagittal plane. Kinematic data of the knee were based on the three respective markers for each leg in the sagittal plane. To provide an asymmetrical marker set and thus more stable real-time marker tracking, two markers were additionally affixed on the middle distal edge of the right footplate and one on the left footplate of the sliding board. Participants were positioned to allow a maximum knee flexion angle of approximately 100° when reaching an in-built physical stop at the proximal end of the board. For all movements, a constant knee angular velocity of 10°/s was attempted. This was practiced during familiarization using real-time graphical feedback for three trials per leg. If knee angular velocity fluctuated by more than 5°/s for consecutive trials of the MRI protocol, participants were verbally reminded to move slower or faster accordingly. Automated instructions were provided throughout the tests based on knee angle and angular velocity calculated from the real-time kinematic data as further described henceforth.

While supine in the Start position (legs fully extended), participants were instructed to flex a specified leg (randomized order) until a stop sign appeared (randomly activated at 35° or 60° knee flexion, but participants told angles were random). Two seconds after stopping, participants were instructed to maintain the position and memorize the knee (target) angle. Eight seconds later, they were instructed to return to the Start position and then 5 s after returning were instructed to reproduce the same angle. Four seconds after stopping, participants were instructed to return to the Start position. This was performed eight times per angle on each leg, resulting in a total of 16 repetitions for the JPS test on each leg. Additionally, knee flexion to the physical stop at ∼100° was performed on eight separate occasions per leg, with the timeframe from 0–65° extracted for brain imaging analysis for a *Flex* condition. The *JPS* and *Flex* conditions were pseudorandomized with a maximum two consecutive repetitions of the same condition and a minimum 7 s between trials. A *Rest* condition (Start position for 15 s) was also included five times at evenly-spaced intervals throughout testing. Thus, the task had a block design, individualized based on kinematic data. We utilized three different experimental conditions: (1) *JPS* condition – active knee flexion during angle reproduction (start/end at onset/cessation of flexion, respectively), (2) *Flex* condition – active knee flexion without angle reproduction (start at onset of flexion and end when reaching 65° knee angle), and (3) *Rest* condition – Start position (start/end at cessation of extension and onset of flexion, respectively). The protocol lasted approximately 40 min, resulting in 1240 whole-brain sets.

The current fMRI-adapted knee JPS test was also assessed for test-retest reliability in our movement laboratory among a separate group of 15 (9 males) asymptomatic persons (mean ± SD: age 25.0 ± 3.1 years, height 1.78 ± 0.09 m, mass 74.4 ± 11.2 kg) who performed the test on two occasions 7 days apart. Reliability was estimated with Intraclass Correlation Coefficients (ICC) and 95% confidence intervals (CI) based on a mean rating (*k* = 10), two-way mixed effects model with absolute agreement and Standard Error of Measurement (SEM), calculated as the mean square error term from the ANOVA, separately for the non-dominant [ICC 3,10 = 0.64 (CI 0.02–0.87), SEM = 0.67°] and dominant leg [ICC 3,10 = 0.78 (CI 0.34–0.93), SEM = 0.86°].

### Image Acquisition

A 3T General Electric MR scanner with a 32-channel head coil was used to acquire the MR images. A T1 structural image was first acquired to create a study-specific template using the following parameters: 180 slices; 1 mm thickness; repetition time 8.2 ms; echo time 3.2 ms; flip angle 12°; field of view 25 × 25 cm. The functional gradient-echo-planar imaging sequence was collected with the following scanning parameters: repetition time = 2000 ms, echo time = 30 ms, flip angle = 80°, field of view = 25 × 25 cm. Thirty-seven transaxial slices with a thickness of 3.4 mm (0.5 mm gap) were acquired in an interleaved order. Ten initial dummy scans were collected and discarded prior to analysis. Test instructions were presented on a computer screen, which were seen *via* a tilted mirror attached to the head coil. The computer parallel port was used to detect the trigger output signal from the MR scanner to synchronize kinematic data with fMRI data in later analyses.

### Data Processing and Analysis

Motion capture data were exported to Visual3D software (v.5.02.19, C-Motion Inc., Germantown, MD, United States) and filtered with a 6 Hz fourth-order low-pass zero-lag Butterworth filter. Automated scripts set events based on knee angles and knee angular velocities in the sagittal plane. Target and reproduction angles were extracted 2 s after cessation of flexion during the respective phases. All events were checked visually by the lead researcher and adjusted if deemed incorrect. No data were removed from analyses. Data were exported to IBM SPSS Statistics for Windows, version 25 (IBM Corp., Armonk, N.Y., United States) in which all statistical analyses for knee JPS outcome measures, participant characteristics and patient-reported outcomes were performed.

SPM12 software (Wellcome Department of Cognitive Neurology, London, United Kingdom) run under MATLAB R 2016 b (MathWorks, Inc., Natick, MA, United States) was used for automated batching, pre-processing and data analysis. SPM was used for visualization of statistical maps and MarsBaR 0.44 (Brett et al., 2002) was used to calculate the percentage of BOLD signal change. Data were pre-processed in the following way: slice timing correction (interleaved order, first image set to reference slice), movement correction by unwarping and realigning all subsequent scans to the first image, co-registration of the mean functional image set and the structural T1 image set, segmentation of the co-registered structural image, normalization to a sample-specific template based on white and gray matter segments from the segmented, co-registered, structural image [using DARTEL (Ashburner, 2007)] and affine alignment to Montreal Neurological Institute (MNI) standard space and smoothing with an 8-mm FWHM Gaussian kernel. The final voxel size was 2 × 2 × 2 mm.

### Statistical Analyses

The ACLR group was subdivided into those with right-side (R-ACLR) and left-side (L-ACLR) reconstruction. Sex distribution between groups was analyzed with a Chi-square test. Age and current activity level (Tegner current and Marx activity) between all groups, as well as pre-injury activity level of the ACLR groups to current activity level of CTRL, were analyzed with Kruskal-Wallis tests and Dunn-Bonferroni *post hoc* tests for significant results. The questionnaires TSK, IKDC and Lysholm Scale were compared between ACLR groups with Mann-Whitney *U* tests. A One-way analysis of variance (ANOVA) compared body height and mass between all groups, as well as months since reconstruction between ACLR groups.

Between-group comparisons for kinematic data and brain response were made between the injured leg of ACLR and the matched leg of CTRL. Thus, the R-ACLR group was compared to controls only when the right leg was active for each group and the L-ACLR group were compared to controls only when the left leg was active for each group. Knee JPS error was defined in degrees as the absolute difference [absolute error (AE)] between the target and reproduction angles of the knee for each repetition of the JPS test. Mean AE was calculated for each leg by pooling the 40° and 65° angle conditions for each participant. Outliers in the data set were assessed for eligibility at group level, but none were removed due to a lack of evidence to the contrary. Shapiro-Wilk tests of normality and analysis of distribution graphs confirmed non-normally distributed group-level data. Knee JPS data were thus log transformed and were subsequently considered normally distributed according to further Shapiro-Wilk tests. Independent samples t-tests were used to compare the log transformed knee JPS AE between groups. Significance levels were set *a priori* (α = 0.05).

Brain imaging data for each angle condition were pooled so that each participant contributed with data from 16 *JPS* trials per leg. The first-order (single-subject) analyses were set up by including the experimental conditions as regressors of interest in the general linear model, convolved with the hemodynamic response function. Six realignment parameters (head rotations and translations) were included as covariates of no interest to account for movement artifacts. The following contrasts were set up for each participant: (1) (*JPS* > *Rest*) and (2) (*Flex* > *Rest*). Second-order (group) analyses were based on flexible factorial models (Gläscher and Gitelman, 2008) of the (*JPS* > *Rest*) and (*Flex* > *Rest*) contrasts from each participant, including participant, group (two levels, R-ACLR or L-ACLR and CTRL), condition (two levels, *JPS* and *Flex*) and the interaction (group × condition) as factors. Separate analyses of the conditions used in the flexible factorial design (*JPS* > *Rest*) and (*Flex* > *Rest*) were also performed to investigate activation patterns in comparison to *Rest*. These analyses are included as supplementary material. The main effect from condition [(*JPS* > *Rest*) > (*Flex* > *Rest*)] and the interaction (group × condition) was analyzed [family-wise error (FWE) rate corrected, 0.05; voxel limit 15]. Brain regions were defined and labeled according to the MNI coordinates, which relate to the peak activity within the cluster, using automated anatomic labeling in SPM (Tzourio-Mazoyer et al., 2002). Brain regions that showed significant activation for any of these analyses were further analyzed by calculating the percentage of BOLD signal change during the *JPS* condition (i.e., the original beta values) in the significant region, compared to the overall mean brain activity of the session. The percentage of BOLD change values were exported to SPSS where Spearman’s rho was used to analyze correlations of participant mean values between percentage of BOLD change and JPS errors.

## Results

**FIGURE 2:**
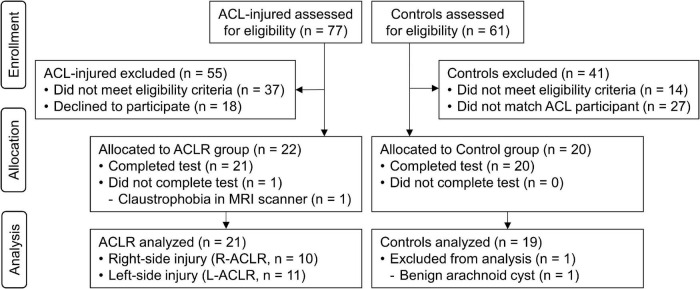
Flow diagram illustrating the recruitment process.

Of 77 persons with ACL injury and 61 potential controls who expressed an interest in participating, 55 and 14, respectively, were considered ineligible due to either another existing injury, too low physical activity level, left leg dominance for controls, older age, or a combination of those factors. Thus, 22 individuals with ACLR and 47 asymptomatic controls were considered eligible for the study. To ensure matching of characteristics between groups, controls were invited to participate only after a matching ACLR participant had completed testing. One ACLR participant did not complete the fMRI procedure due to claustrophobic feelings in the MRI scanner. Despite completing testing, one asymptomatic control was not included in the analyses due to the presence of a benign arachnoid cyst, unknown prior to participation, which would have confounded brain imaging analyses. Thus, 10 R-ACLR, 11 L-ACLR and 19 CTRL completed testing and were included in the analyses (see Figure 2 for a flow diagram of the recruitment process and Table 1 for group characteristics). Due to a technical issue, one L-ACLR participant completed a shortened protocol of 12, instead of 16, repetitions per leg. Also, due to slow performance of the test, one participant from each group performed one less repetition on both legs. Current activity level of CTRL was significantly lower than pre-injury level of R-ACLR (*P* = 0.010). No groups differed significantly with regard to sex, age, height, weight or the remaining patient-report outcome measures. Months since reconstruction did not differ significantly between the ACLR groups. For the knee JPS test, no statistically significant differences in errors were seen between either the left reconstructed/non-dominant leg of L-ACLR [median (Mdn) 5.10° (Q1 4.04°, Q3 6.82°)] and CTRL [Mdn 4.17° (Q1 2.89°, Q3 4.95°)], respectively, or between the right reconstructed/dominant leg of R-ACLR [Mdn 4.89° (Q1 3.54°, Q3 6.85°)] and CTRL [4.55° (Q1 3.64°, Q3 6.56°)], respectively.

**TABLE 1:** Participant characteristics of the study groups.

	R-ACLR	L-ACLR	CTRL
Participants, n	10	11	19
Age, y, mean (SD)	24.8 (4.2)	28.2 (4.7)	27.1 (4.6)
Male:female, n	4:6	4:7	7:12
Months since reconstruction, mean (SD)	20.0 (9.7)	28.5 (18.6)	–
Body height, m, mean (SD)	1.72 (0.09)	1.73 (0.09)	1.75 (0.08)
Body mass, kg, mean (SD)	72.6 (7.8)	72.1 (11.0)	73.1 (9.9)
**Patient-reported outcome scales, median (IQR)**			
IKDC 2000,% of maximum	77.6 (14.1)	77.0 (15.0)	–
Lysholm score	86.0 (12.5)	86.0 (5.0)	–
Marx activity score	12.0 (6.5)	10.0 (7.0)	11.0 (7.0)
Tegner pre-injury score	8.5 (1.2)^†^,^‡^	8.0 (2.0)	–
Tegner current score	5.5 (2.2)	7.0 (4.0)	6.0 (4.0)
TSK score	36.5 (11.5)	33.0 (5.0)	–
Injury mechanism, non-contact:contact, n	8:2	11:0	–
Leg dominance, right:left, n	10:0	8:3	19:0
**Injury activity, n**			
Soccer	4	2	
Downhill skiing	2	4	
Martial arts	0	2	
Basketball	0	1	
Dancing	1	0	
Floorball	1	0	
Gymnastics	0	1	
Rugby	1	0	
Snowboard	1	0	
Volleyball	0	1	

*^†^Significantly greater than R-ACLR Tegner current score (P = 0.011).*

*^‡^Significantly greater than CTRL Tegner current score (P = 0.010).*

*CTRL, asymptomatic control group; IKDC 2000, International Knee Documentation Committee Subjective Knee Form; IQR, interquartile range; L-ACLR, left-side anterior cruciate ligament-reconstructed group; R-ACLR, right-side anterior cruciate ligament-reconstructed group; TSK, Tampa Scale of Kinesiophobia.*

### Brain Response During the Knee Joint Position Sense Test

**FIGURE 3:**
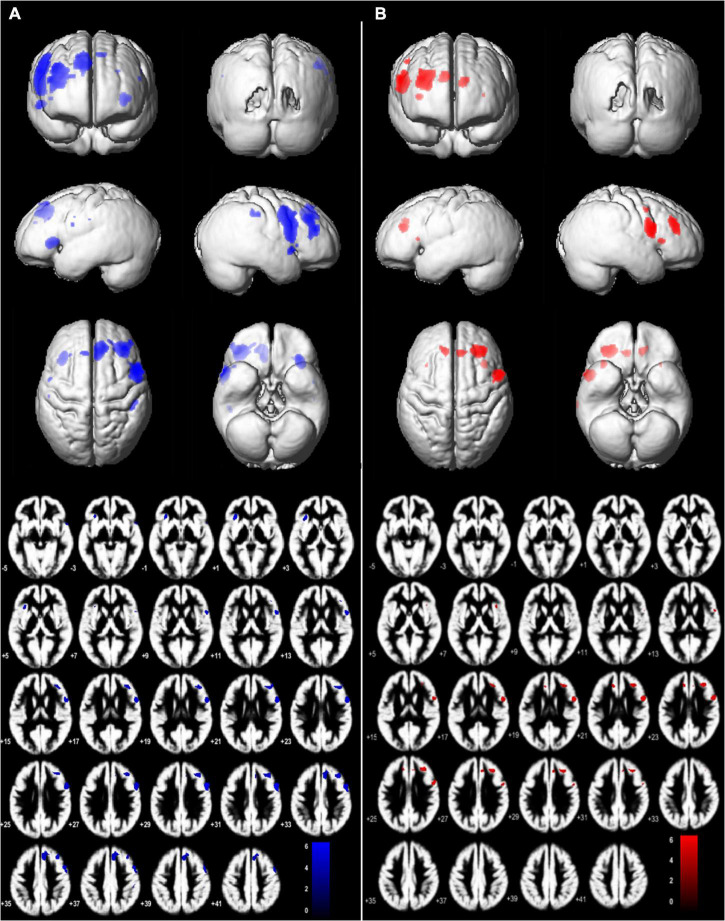
Brain regions with significant main effect condition in (*JPS* > *Rest*) > (*Flex* > *Rest*), *P* = 0.05, family-wise corrected, voxel limit 15. Slices –5:2:38 mm (MNI) in inferior-superior direction are shown. Group mean brains (Dartel) are used for the illustration for **(A)** left-side analyses – L-ACLR moving their injured left leg and CTRL moving their left non-dominant leg, and **(B)** right-side analyses – R-ACLR moving their right injured leg and CTRL moving their right non-dominant leg.

**FIGURE 4:**
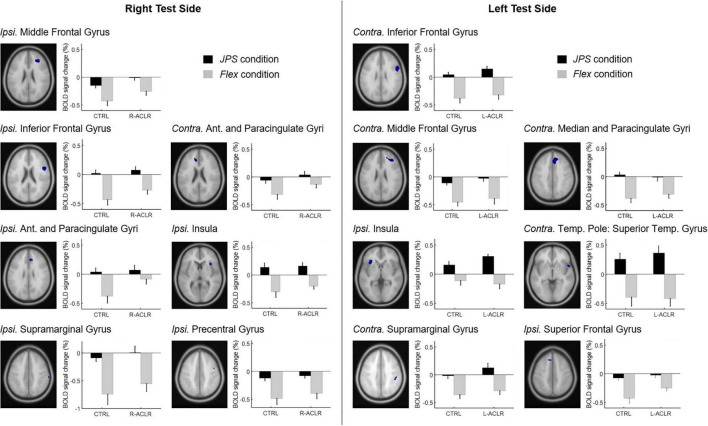
BOLD signal change (%) during angle reproduction of the JPS test for the brain regions with a significant main effect from condition [(*JPS* > *Rest*) > (*Flex* > *Rest*)]. Ant., Anterior; *Contra.*, contralateral; CTRL, asymptomatic control group; *Flex*, flex condition; *Ipsi.*, ipsilateral; JPS, joint position sense condition; L-ACLR, left side anterior cruciate ligament-reconstructed group; R-ACL, right-side anterior cruciate ligament-reconstructed group; Temp., Temporal.

The JPS condition evoked significantly greater BOLD response (*P* = 0.05, FWE corrected; voxel limit 15) in seven brain regions for each leg compared to the Flex condition without a JPS task. These included prefrontal regions, the precentral gyrus, cingulate gyri and insula (see Figures 3, 4 and Table 2 for all significant regions with voxel extent, exact statistics, and MNI coordinates).

**TABLE 2:** Brain regions with significantly greater bold response during *JPS* than *Flex* [(*JPS* > *Rest*)] > (*Flex* > *Rest*)]^†^.

Test side	Brain regions	Voxel #	*P*	Z max	MNI coordinates		
					*X*	*Y*	*Z*
Left	*Contra.* Inferior frontal gyrus	1608	0.000	6.15	51	9	30
	*Contra.* Middle frontal gyrus	1049	0.000	5.33	36	30	33
	*Contra.* Median and Paracingulate gyri	864	0.000	5.29	8	29	36
	*Ipsi.* Insula^‡^	330	0.001	5.63	−32	24	2
	*Contra.* Temp. Pole: Superior temp. Gyrus	97	0.008	5.17	54	11	−5
	*Contra.* Supramarginal gyrus	84	0.010	4.60	48	−29	38
	*Ipsi.* Superior frontal gyrus	43	0.018	4.57	−9	29	47
Right	*Ipsi.* Middle frontal gyrus	666	0.000	5.51	29	33	26
	*Ipsi.* Inferior frontal gyrus	596	0.000	5.76	51	5	20
	*Contra.* Ant. and Paracingulate gyri	152	0.005	4.98	−9	33	21
	*Ipsi.* Ant. and Paracingulate gyri^‡^	135	0.006	4.67	8	33	26
	*Ipsi.* Insula	61	0.015	4.70	35	20	6
	*Contra.* Supramarginal gyrus^‡^	15	0.031	4.70	65	−26	42
	*Ipsi.* Precentral gyrus	15	0.031	4.42	47	0	42

*^†^Seven brain regions for each test side showed significantly greater BOLD response during the JPS condition compared with the Flex condition across groups.*

*^‡^Significant correlation between knee JPS AE and BOLD percentage change. For left test side n = 30 (L-ACLR 11 and CTRL 19), right test side n = 29 (R-ACLR 10 and CTRL 19).*

*Test side: the leg that was active during the JPS test; Voxel #: indicates number of activated voxels in this cluster; P: 0.05 family wise error rate corrected (cluster level); Z max: Z-score of the voxel with the highest activity for main effect from condition; MNI: voxel with the highest activity in MNI-space. Ant., anterior; Contra., contralateral; CTRL, asymptomatic control group; Flex, flex condition; Ipsi., ipsilateral; JPS, joint position sense condition; L-ACLR, left-side anterior cruciate ligament-reconstructed group; MNI, Montreal Neurological Institute; Rest, rest condition; R-ACLR, right-side anterior cruciate ligament-reconstructed group; Temp., temporal.*

### Between-Group Comparisons of Brain Response During the Knee Joint Position Sense Test

No significant between-group differences were found on the corrected level (FWE 0.05).

### Correlations Between Brain Response and Knee Joint Position Sense Errors

**FIGURE 5:**
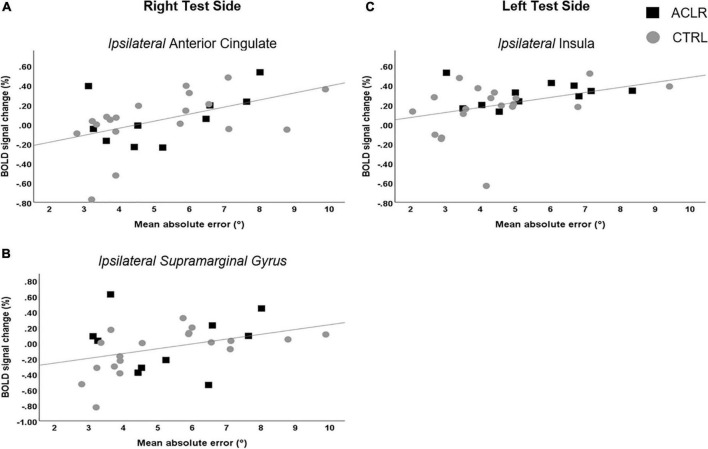
Scatter plots illustrating the significant correlations between mean knee joint position sense absolute errors and simultaneous percentage change in BOLD response for the right test side **(A,B)** and the left test side **(C)**.

Correlation analyses were performed for the seven regions per test side that were found to have significantly greater BOLD response during the JPS condition compared to the Flex condition (see Table 2 for a list of the regions). When performing the test with the right leg (R-ACL and CTRL, *n* = 29), significant positive correlations were found between JPS errors and BOLD signal percentage change (i.e., greater JPS AE correlated with greater BOLD response) in the ipsilateral anterior cingulate (*r* = 0.476, *P* = 0.009; Figure 5A) as well as the ipsilateral supramarginal gyrus (*r* = 0.395, *P* = 0.034; Figure 5B). Close to significance was also the ipsilateral middle frontal gyrus (*r* = 0.364, *P* = 0.052). For the left leg (L-ACLR and CTRL, *n* = 30), a significant positive correlation was found for the ipsilateral insula (*r* = 0.474, *P* = 0.008; Figure 5C).

## Discussion

We aimed to characterize brain response during a knee JPS test among asymptomatic controls and individuals with ACL reconstruction. We further investigated whether brain response would differ between groups and whether brain response would correlate with knee JPS errors. Our hypothesis that our knee JPS test would evoke greater response in somatosensory and motor cortices compared to simple knee flexion was confirmed by observations of greater response during angle reproduction in, for example, the precentral gyrus, middle frontal gyrus, insula and cingulate gyri. The lack of significant differences between individuals with ACL reconstruction compared to asymptomatic controls was, however, contradictory to our hypothesis. Greater knee JPS errors correlated with BOLD response in the insula, anterior cingulate and supramarginal gyrus, thus confirming our hypothesis of correlations between brain response and knee JPS errors.

### Brain Response During a Knee Proprioception Test

Our knee JPS test evoked response in the *ipsilateral* precentral gyrus for the right test side and cingulate gyri for both test sides. Response in these regions has also been observed among asymptomatic individuals during active knee flexion tasks of JPS (Callaghan et al., 2012) and force matching (Grooms et al., 2021). Response in the right middle frontal gyrus was seen for both test sides and has been previously associated with switching between external and internal focus of attention (Japee et al., 2015), relevant for our JPS task where the focus of attention changes from external instructions on a screen to internal sensations related to proprioception. Also common for both test sides was recruitment of the *ipsilateral* insula, previously associated with sensorimotor processes such as active and passive stepping motions (Jaeger et al., 2014). This finding also aligns with the body image and body schema concepts of body representations (Head and Holmes, 1911), in which current understandings attribute the insula with conscious perceptual representation of the body and memory (Dijkerman and De Haan, 2007). The insula and anterior cingulate are further thought to play a key role in interoception, a term originally introduced by Sherrington (1906) to describe visceral sensations, but now often used as a broader term encompassing the subjective experience of the body state (Ceunen et al., 2016) and even proprioception (Hillier et al., 2015). In fact damage to the insula due to stroke has been associated with poor position sense of the upper limbs (Findlater et al., 2016). The same study also found similar associations for the inferior frontal and superior temporal gyri, two areas that were activated during our JPS test in the current study. Thus, our findings add to previous evidence of distributed neural networks involved when processing proprioceptive tasks and expand these to those associated with the lower limbs. Interestingly, 11 of the 14 regions with significantly greater BOLD response during the *JPS* condition compared with the *Flex* condition were of the right hemisphere (Table 2). A general dominance of the right hemisphere for proprioception in the lower limbs is supported by previous findings for a foot position matching task among healthy individuals who were right hand and foot dominant, suggesting a role for position sense of the right parietal and frontal cortex (Iandolo et al., 2018). In fact, research into body representations further seems to implicate the right hemisphere with kinesthetic sensations (Naito et al., 2016). If future research confirms these results, the traditional network of brain regions believed to be associated with proprioception of the lower limbs should thus be extended and include an emphasis on the right hemisphere. To improve our understanding further, extended mapping of associated neural networks is required.

### Brain Response of Individuals With Anterior Cruciate Ligament Reconstruction Compared to Matched Controls

There were no significant between-group differences for brain response nor for knee JPS errors. Previous research investigating knee JPS and somatosensory evoked potentials of individuals 18 months after surgical reconstruction of the ACL also found a lack of difference compared with controls as evidence of sensory neurone regeneration (Ochi et al., 1999). The original version of the current supine knee JPS test also did not detect any significant differences in errors for a separate ACLR group approximately 2 years after surgical reconstruction compared with matched athletes (Strong et al., 2021b). In that study, less-active controls instead showed significantly greater JPS errors compared to the ACLR group, suggesting that activity level is a more important factor in this context. It is therefore possible that deficits in proprioception were not present among the individuals of our ACLR group, who were active and participated on average 23 months after surgical reconstruction. Additionally, a recent meta-analysis found that only knee JPS tests with passive rather than active movements differentiate between ACL-injured knees and those of asymptomatic controls (Strong et al., 2021a). The active movements of the current test, which also incorporated the hip, may further have contributed to the lack of between-group difference seen here. The target angles of 40° and 65° knee flexion used in our JPS test both lie close to the mid-range of motion for the joint. These angles may not have been optimal for elucidating differences between groups where joint receptors are the focus of investigation, given that they are believed to play a more predominant role toward the limits of joint rotation (Proske and Chen, 2021). It is also possible that small group sizes (due to separating ACLR into right and left leg analyses), as well as the contrast to such a similar movement, may have reduced the sensitivity of our analysis.

### Correlations Between Brain Response and Knee Joint Position Sense Errors

Our results showed that greater knee JPS errors, i.e., poorer knee proprioceptive acuity, was associated with greater brain response in the ipsilateral insula for the left test side, as well as the ipsilateral anterior, paracingulate and supramarginal gyri for the right test side. These results are line with the Embodied Predictive Interoception Coding (EPIC) model proposed by Barrett and Simons (Barrett and Simmons, 2015), which describes the process of active inference in interoception. Part of this model describes the role of the mid- and posterior insula in computing and transmitting prediction errors as well as the integration of other agranular visceromotor cortices such as the cingulate cortex in this process. This model may thus be relevant for proprioceptive tasks. The importance of the insula to position sense is further supported by findings previously mentioned here for the upper limbs following stroke, whereby lesions in this region were associated with greater errors when attempting to actively move the unaffected arm to the mirror-matched position of the passively moved contralateral arm (Findlater et al., 2016). Associated response for the right supramarginal gyrus is supported by previous findings of greater response during a proprioceptive task of force matching at the knee among asymptomatic females (Grooms et al., 2021).

### Limitations of the Study

A limitation of our study was the additional task of attempting a constant knee angular velocity, although this was similar for the contrast *Flex* condition. Further, the active movement of the whole lower limb meant that proprioceptive feedback was not isolated to the knee, but also incorporated the hip. Although this is more similar to everyday activities and thus may enhance the ecological validity of the task compared with a single-joint movement, compensations at the hip joint for potential deficits at the knee are possible. Despite the active task, head movement (range 0.14 – 0.88 mm) did not confound brain imaging analyses. Due to challenges in recruiting participants, we included ACLR individuals who had injured either knee, but a control group with only right-side dominance. Comparisons were thus made to the non-dominant and dominant legs of CTRL for the L-ACLR and R-ACLR groups, respectively. Evidence regarding whether leg dominance influences knee proprioception among healthy controls has, however, shown both positive (Azevedo et al., 2020) and negative (Cug et al., 2016; Galamb et al., 2018) findings. Defining leg dominance for an unfamiliar task is also complicated given evidence of task specificity (Van Melick et al., 2017). It is thus unclear whether leg dominance influenced our results. Further, having to split the ACL participants into two groups meant that the sample sizes for between-groups comparisons were not in line with our aims. We acknowledge this as an important limitation of the current study. Future studies with a greater number of and more homogenous participants are likely required to further elucidate potential group differences. Additionally, both sexes were represented in each of our groups. Two similar fMRI studies have, however, indicated potentially different functional brain connectivity between males (Diekfuss et al., 2020) and females (Diekfuss et al., 2019) who later suffered an ACL injury. Our eligibility criteria required a minimum physical activity level score of four according to the Tegner activity scale. Although we matched current activity level between groups, a sub-analysis using Wilcoxon Signed Ranks tests compared pre-injury and current activity levels within the ACLR groups and found that R-ACLR had significantly reduced their activity level (*P* = 0.011), but the level was not significantly changed among L-ACLR. A difference in activity level change from pre- to post-injury was thus a potentially confounding factor in our analyses. To explore this further, a Mann-Whitney U test to compare change in activity level between the groups found no significant between-group difference. A significant reduction in activity level following ACL injury is, however, considered a useful outcome for defining non-copers (Button et al., 2006). Future studies may thus benefit from analyses that consider coping classification of individuals with ACL injury. To improve the sensitivity of the analyses, future test designs should also consider how to increase the number of brain images during movements which require proprioceptive processing. This could, for example, be done by increasing the duration of the proprioceptive element of such tasks.

## Conclusion

Our paradigm found greater BOLD response for a number of brain regions which have previously been associated with processes that can be linked to proprioception. These results thus indicate that the experimental design was successful in recruiting brain regions involved in proprioception and adds valuable information regarding central processing of such tasks. The lack of differences between groups adds to the mixed evidence of proprioceptive deficits among individuals nearly 2 years post-ACLR. The small sample sizes as a result of splitting the ACLR group into left and right-side injuries may, however, have been a contributing factor to the lack of significance. The significant correlations between knee JPS error and activation in some brain regions further indicates that demands were placed on the proprioceptive acuity of the participants. The novel integration of kinematics with fMRI thus provided added value to the paradigm by providing behavioral data as well as specific time frames for extraction of brain images to isolate such processes. Our unique paradigm demonstrates a method to expand these findings and provide further insights into brain response to proprioception at the knee and other joints as well as among different populations.

## Data Availability Statement

The raw data supporting the conclusions of this article will be made available by the authors, without undue reservation.

## Ethics Statement

The studies involving human participants were reviewed and approved by Regionala etikprövningsnämnden i Umeå. The patients/participants provided their written informed consent to participate in this study.

## Author Contributions

JS created the integrated software used for data collection. AS and CH recruited participants. AS and JS performed data collection. AS and HG processed and analyzed the kinematic and brain imaging data. AS, HG, and C-JB performed statistical analyses. AS wrote the first draft of the manuscript. HG and JS wrote sections of the manuscript. All authors contributed to the conception and design of the study and contributed to manuscript revision, read, and approved the submitted version.

## Conflict of Interest

The authors declare that the research was conducted in the absence of any commercial or financial relationships that could be construed as a potential conflict of interest.

## Publisher’s Note

All claims expressed in this article are solely those of the authors and do not necessarily represent those of their affiliated organizations, or those of the publisher, the editors and the reviewers. Any product that may be evaluated in this article, or claim that may be made by its manufacturer, is not guaranteed or endorsed by the publisher.
