# Supplementary material for: Brain Response to a Knee Proprioception Task Among Persons With Anterior Cruciate Ligament Reconstruction and Controls
Source: Front Hum Neurosci. 2022 Mar 22;16:841874. doi: 10.3389/fnhum.2022.841874 (PMC8980265; doi:10.3389/fnhum.2022.841874)
Supplement: Supplementary file 1 [file Data_Sheet_1.DOCX]

Supplementary Material

# Summary of supplementary results

The supplementary results contain further analyses of the *Flex* and *JPS* conditions. The aim of the supplementary analysis is to confirm the validity of the contrasts regarding the research questions (*JPS > Rest*) > (*Flex* > *Rest*) and to verify that the conditions irrespectively of each other activate expected brain regions. The conditions [*JPS* > *Rest*] and [*Flex* > *Rest*] provide similar functional brain responses, and, as expected, during the *JPS* condition a larger brain network is activated than during the *Flex* condition. Activation is most intense on the contralateral side, but bilateral activation occurs, as expected (see Supplementary Table 1 and 2).

**Supplementary Table 1.** Brain regions with significantly greater BOLD response during the *JPS* condition compared to resting [*JPS* > *Rest*]. For left test side n = 30 (CTRL 19 and L-ACLR 11) and for right test side n = 29 (CTRL 19 and R-ACLR 10). Statistics: familywise correction p = 0.05, cluster threshold level 15.

| Test side | Brain regions | Voxel # | *P* | Z max | MNI coordinate | | |
| --- | --- | --- | --- | --- | --- | --- | --- |
|  |  |  |  |  | *X        Y       Z* | | |
| Left | Right insula | 1699 | .000 | 6.39 | 45 | 5 | 6 |
|  | Left insula | 1563 | .000 | 6.32 | -45 | 0 | 6 |
|  | Right supramarginal gyrus | 429 | .000 | 6.27 | 48 | -32 | 26 |
|  | Left middle occipital cortex | 41 | .001 | 5.42 | -18 | -101 | 2 |
|  | Right precuneus | 1273 | .001 | 5.38 | 12 | -45 | 68 |
|  | Left rolandic operculum | 43 | .009 | 4.83 | -44 | -32 | 21 |
|  | Left precuneus | 88 | .009 | 4.81 | -11 | -47 | 66 |
| Right | Left precuneus | 41360 | .000 | 7.56 | -8 | -42 | 65 |
|  | Left superior temporal gyrus | 513 | .000 | 5.99 | -47 | -2 | 6 |
|  | Right calcarine sulcus | 227 | .000 | 5.81 | 8 | -93 | -14 |
|  | Left rolandic operculum | 235 | .000 | 5.81 | -41 | -32 | 20 |
|  | Left middle cingulum | 590 | .001 | 5.32 | -2 | -5 | 48 |
|  | Right insula | 82 | .005 | 4.92 | 36 | 17 | 8 |
|  | Left putamen | 226 | .007 | 4.84 | -29 | 0 | 8 |

*Contra*, contralateral; CTRL, asymptomatic control group; *Ipsi*, ipsilateral; *JPS*, joint position sense condition; L-ACLR, left-side anterior cruciate ligament-reconstructed group; MNI, Montreal Neurological Institute; *Rest*, rest condition; R-ACLR, right-side anterior cruciate ligament-reconstructed group.

**Supplementary Table 2.** Brain regions with significantly greater BOLD response during the *Flex* condition compared to resting [*Flex* > *Rest*]. For left test side n = 30 (CTRL 19 and L-ACLR 11) and for right test side n = 29 (CTRL 19 and R-ACLR 10). Statistics: familywise correction p= 0.05, cluster threshold level 15

| Test side | Brain regions | Voxel # | *P* | Z max | MNI coordinate | | |
| --- | --- | --- | --- | --- | --- | --- | --- |
|  |  |  |  |  | *X        Y       Z* | | |
| Left | Left middle occipital cortex | 92 | .000 | 6.15 | -18 | -101 | 2 |
|  | Right precuneus | 1928 | .000 | 5.89 | 11 | -45 | 66 |
|  | Left rolandic operculum | 340 | .000 | 5.87 | -47 | -2 | 6 |
|  | Right rolandic operculum | 204 | .000 | 5.82 | 45 | -30 | 23 |
|  | Right insula | 150 | .000 | 5.56 | 45 | 3 | 5 |
|  | Left rolandic operculum | 92 | .001 | 5.34 | -44 | -32 | 21 |
|  | Right superior occipital cortex | 41 | .003 | 5.04 | 21 | -99 | 6 |
|  | Cerebellum | 249 | .003 | 5.04 | -3 | -48 | -15 |
| Right | Left precuneus | 33960 | .000 | inf | -8 | -44 | 65 |
|  | Left rolandic operculum | 154 | .000 | 5.95 | -41 | -32 | 20 |
|  | Left rolandic operculum | 91 | .001 | 5.37 | -47 | -2 | 5 |

*Contra*, contralateral; CTRL, asymptomatic control group; *Ipsi*, ipsilateral; *JPS*, joint position sense condition; L-ACLR, left-side anterior cruciate ligament-reconstructed group; MNI, Montreal Neurological Institute; *Rest*, rest condition; R-ACLR, right-side anterior cruciate ligament-reconstructed group.

# Comparisons *JPS*-*Rest* and *Flex*-*Rest*

Supplementary Figure 1 illustrates similarities and differences between brain activation during the *JPS*>*Rest* and *Flex*>*Rest* test conditions. The activation is mainly contralateral to the engaged leg, but bilateral activation occurs as well. The activation pattern is rather similar between the two conditions, but note that more extensive brain activation occurs during *JPS*>*Rest* than during *Flex*> *Rest*, as expected.


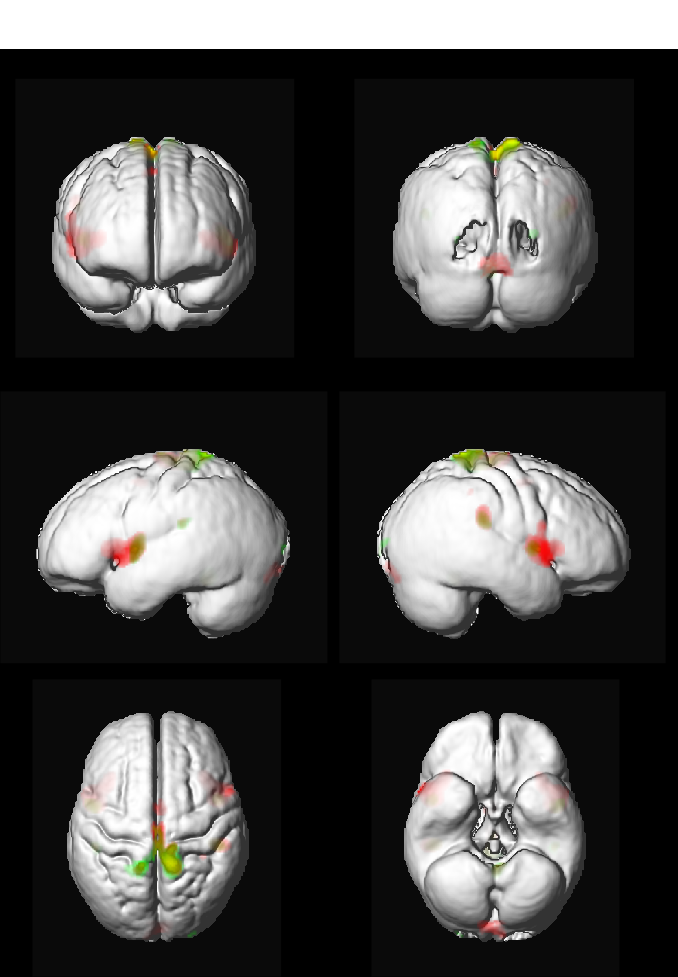

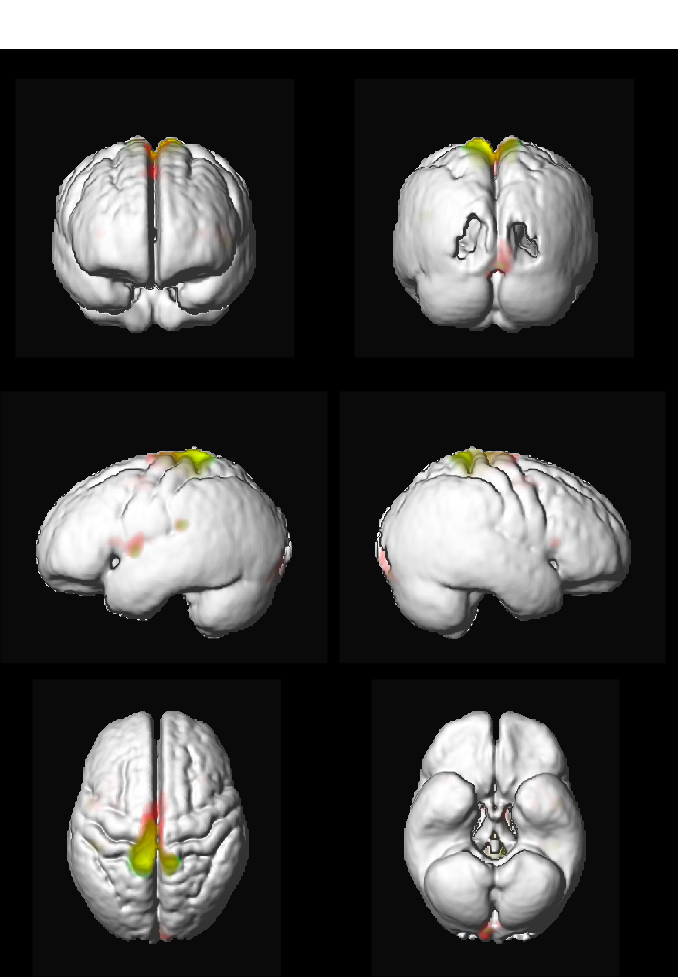


**Supplementary Figure 1.** Left-knee injured ACL individuals and Controls moving their left knee (left image) and Right-knee injured ACL individuals and Controls moving their right knee (right image). The two conditions *Flex*>*Rest* and *JPS*>*Rest* are illustrated. Red = *JPS*>*Rest*, Green = *Flex*-*Rest*, Yellow = common areas (activated during both conditions). The color intensity illustrates the activation intensity.
